# Rethinking the electronic health record through the quadruple aim: time to align its value with the health system

**DOI:** 10.1186/s12911-020-1048-9

**Published:** 2020-02-17

**Authors:** Hassane Alami, Pascale Lehoux, Marie-Pierre Gagnon, Jean-Paul Fortin, Richard Fleet, Mohamed Ali Ag Ahmed

**Affiliations:** 1 Center for Public Health Research (CreSP), Université de Montréal, P.O. Box 6128, Branch Centre-Ville, Montreal, Quebec H3C 3J7 Canada; 2 Institute for Excellence in Health and Social Services (INESSS), Montreal, QC Canada; 3 Department of Health Management, Evaluation and Policy, École de santé publique de l’Université de Montréal, Montreal, Quebec Canada; 4 Research Center on Healthcare and Services in Primary Care, Institute of Health and Social Services in Primary Care, Université Laval, Quebec City, Quebec Canada; 5 Faculty of Nursing Science, Université Laval, Quebec City, Canada; 6 Department of Social and Preventive Medicine, Faculty of Medicine, Université Laval, Quebec City, Quebec Canada; 7 Department of Family Medicine and Emergency Medicine, Faculty of Medicine, Université Laval, Quebec City, Quebec Canada; 8 Research Chair in Emergency Medicine Université Laval-CHAU Hôtel-Dieu de Lévis, Lévis, Quebec Canada; 9 Research Chair on Chronic Diseases in Primary Care, Université de Sherbrooke, Chicoutimi, QC Canada

**Keywords:** Electronic health records, Quadruple aim, Value, Clinic, Burden, Health care delivery, Health system

## Abstract

Electronic health records (EHRs) are considered as a powerful lever for enabling value-based health systems. However, many challenges to their use persist and some of their unintended negative impacts are increasingly well documented, including the deterioration of work conditions and quality, and increased dissatisfaction of health care providers. The “quadruple aim” consists of improving population health as well as patient and provider experience while reducing costs. Based on this approach, improving the quality of work and well-being of health care providers could help rethinking the implementation of EHRs and also other information technology-based tools and systems, while creating more value for patients, organizations and health systems.

## Introduction

Recent studies report that electronic health records (EHRs) have come to play an important role in the deterioration of work conditions for health care providers. This situation is mainly attributed to the tedious and time-consuming workload imposed by data entry for administrative and billing purposes, constraints related to inappropriate interfaces and ergonomics, and EHR interoperability issues. These elements have been associated with increased frustration, dissatisfaction, stress, and exhaustion of health care providers [1–3].

These consequences enter in contradiction with the quadruple aim, which suggests that the well-being of health care providers is essential to any strategy that seeks to improve the quality of care, including patient experiences. In order to show how the quadruple aim can help to rethink how EHRs are designed and implemented, this paper clarifies the lessons that can be drawn from considering the unintended consequences of information technology-based tools and systems.

### Unintended consequences of information technology-based tools and systems

In our analysis of 10 major projects in Quebec (Canada), we examined the unintended consequences of Information Technology (IT) solutions in healthcare. It showed that IT solutions, which often interface with EHRs, could contribute to the deterioration of health care providers’ work conditions in different, but interconnected ways [4]:

- **Decreased contact and communication time:** when clinical consultations are more technology-oriented, a sense of depersonalization of the patient-clinician relationship emerges. Technology also creates a feeling of isolation for some health care providers, particularly because of reduced contact time and informal “corridor” discussions with colleagues and other partner teams.
- **Misalignment of technology and the clinical context:** technology providers may prioritize major clinical-administrative scenarios that are not always adapted to the specificity of practice and organization of services in hospitals. Health care providers thus have to align with the “technology-driven” scenarios. By questioning their autonomy and decision-making capacity, IT solutions may push health care providers to feel that they are at the service of technology. This rigidity is also perceived as limiting health care providers’ opportunities for innovation, inventiveness and creativity in their practice.
- **Technology as a control tool:** the possibility of using IT solutions as a means of controlling their activity is seen by health care providers as a challenge to their agency within the organization (due to the “asymmetry of information” upon which such systems are based), therefore of their autonomy of practice.
- **Anxiety and stress:** in several situations, IT solutions may be experienced as a burden. The dysfunction and/or rigidity of the technology engender situations of frustration and stress, even discouragement for health care providers (e.g., false alarms, configuration problems, disconnection of systems after a period of inactivity). The “alert fatigue” phenomenon may lead some health care providers to ignore alerts or disable alarm systems, which may have dramatic consequences for the patient.
- **Cognitive overload:** the manipulation of large amounts of data and information lead to cognitive overload, exhaustion and health care providers’ sense of ineffectiveness.
- **Interoperability:** the parallel utilisation of various non-integrated technological applications (e.g., doctor’s records, patient’s records, pharmacist’s records) may force health care providers to enter the same information several times (a form of “human interoperability”) or to search for information dispersed in different systems.

These findings, which were observed across several IT-based tools and systems deployed in Quebec over several years (1994–2015) and also in other contexts [5–7], are in line with recent studies showing that EHRs have important consequences on clinical practice. In the USA, a study on ambulatory care reports that physicians spend almost 50% of their time on EHRs and office work while only a third of their time is devoted to clinical work [8]. In a medium-sized hospital, primary care physicians would spend 44% of their time on clerical work, and only 24% on communication and direct clinical contact with the patient [9]. Other studies report that the use of the EHR takes about 30% of the consultation time with patients [10, 11].

The research summarized above highlights the extent to which EHRs, and other IT-based tools and systems (e.g., teleconsultation, telemonitoring), could absorb much of health care providers’ attention and energy, to the detriment of interaction and communication with the patient. This not only undermines the human dimension of clinical practice, but patients may feel neglected or abandoned, deteriorating their experience and therefore the quality of the care they receive. Technology-focused consultation could also lead to failures or errors in the diagnosis and/or follow-up of patients (e.g., missing the contextual, psychosocial and emotional cues) [12]. The time is therefore ripe to align the EHR value with the health system.

### Rethinking the EHR through the quadruple aim

Bodenheimer and Sinsky (2014) proposed a set of essential core dimensions in order to optimize the performance of health systems. To the three established dimensions of the “triple aim” -improving the patient experience, improving population health, and reducing costs-, these authors add as a fourth dimension: the improvement of professional lives of health care providers [13]. They point out that professional burnout is associated with patient dissatisfaction, resulting in poor health outcomes and increased costs [14–16]. Thus, to achieve the main objective of improving population health, patient experience and resource utilization, better work conditions and satisfaction of health care providers are also required.

For Bodenheimer and Sinsky, the obligation to meet patients’ legitimate expectations for high quality and empathic services are often not accompanied by conditions and means to achieve them [13]. On the contrary, these requirements have led to increased pressure, frustration, cynicism and suffering due, among other things, to health care providers’ dissatisfaction and sense of under-accomplishment [16, 17]. This could lead to loss of quality in care and services (e.g., lack of empathy, threat to patient safety, less attention to changes in terms of patient’s health conditions), resulting in less satisfied patients with poor health outcomes in the end.

Furthermore, burnout and dissatisfaction of health care providers that are specific to the use of EHRs raise issues of quality and safety of healthcare and services. This problem is an indicator of the dysfunction of the health system and a contributing factor to this dysfunction [18, 19]. In fact, the EHR should not be seen as a mere technological problem, but as a system-level issue. Technology offers an added-value when its use in a real-life context contributes to the objectives of health systems: better patient experience, better health for the population, and reduced costs in a perspective of responsible and sustainable management of resources. In this regard, the quadruple aim offers a compass to guide the design and implementation of technologies with real added-value for patients, health care providers, organizations and health systems.

### Aligning the value of EHRs with the health system

First, the quadruple aim suggests that the funding of IT-based innovation should focus on the needs and realities of the field. It involves looking at evidence of the contribution of technology to improving patient outcomes and experience, as well as health care providers satisfaction, rather than only focusing on administrative and managerial functions, and financial performance. Technology assessment, as part of an overall value chain, should include the improvement of the quality of work conditions and the satisfaction of health care providers in value measures. This perspective requires technological developments that are more focused on practice-related needs and the contexts in which end users (individuals or groups) operate: clinical processes, professional dynamics, and organization of services. It is thus necessary to address issues related to the administrative and cognitive burdens of health care providers as well as the reduction in effectiveness (or feeling of ineffectiveness) that could result from the use of IT-based technology [20].

In this regard, it is important to identify and understand the functionalities that are essential for optimal and patient-centred clinical practice in the organization. Sophisticated technology, with a multitude of functionalities, may not be necessary or relevant in some practice settings. Such optimization remains a challenge, as customizing functionality and adapting technology to the local needs of health care providers and organizations, even patients, could be considered less cost-effective by technology providers [4, 21]. The latter tend to commercialize technologies based on broadly generic “clinical scenarios”, and this, in a logic of “mainstream” technologies [4, 21]. This is where guidelines, incentives and models are needed for technology providers to develop technologies that can be adapted to different contexts. In addition, there is also a need to consider the documentation that health care providers must process or enter: is the EHR implemented to facilitate billing and administrative control, or to provide effective and quality care and services for the patient? Prioritizing either of these two options allows to assess the magnitude of the administrative burden that the health care provider would have to bear (e.g., billing justification) and the technology’s level of user-friendliness (e.g., number of mouse clicks, screen changes, displays, scrolling) [3, 22]. Some tasks may also not require electronic communication (e.g., messages or commands), but rather direct dialogue or conversation between people (e.g., clinician-clinician, clinician-assistant, clinician-patient) [20, 22]. In this regard, regulators still have an important role to play, in particular by revising the documentation and information requirements that must be generated and processed by health care providers: not all elements of care can be captured in the EHR and are not necessarily of clinical added-value [19, 22]. Alternatively, to reduce the time and energy spent on data entry and documentation by health care providers, some authors propose to use virtual scribes based on artificial intelligence [23]. However, it is advisable, once again, to be cautious that another technological layer, which is supposed to solve the problem, will only amplify its root causes [24]. For instance, it may affect certain critical social interactions and dynamics between colleagues and with patients, or disrupts clinical processes and workflows.

Second, the quadruple aim also serves as a reminder that the success of the EHR, primarily as a clinical endeavour, depends in part on the presence of committed and engaged health care providers who think and act positively once they are convinced of the added-value and importance of change. As reported by Sikka et al. (2015), whereas three objectives of the quadruple aim are the *“raison d’être”* of health organizations and systems, the fourth one is an essential condition for achieving them [25]. In this vein, EHRs imply that key stakeholders (e.g., decision-makers, managers, health care providers, patients, technology providers) adopt a participatory and transparent co-construction approach. In other words, they must develop a shared vision of the objectives and scope of the project, but also of the nature and extent of changes and adaptations required, and of the efforts needed to achieve them. So far, decision-makers and promoters of technology projects have tended to attribute failures to “resistance to change” on the part of recipients who “illogically” persist in keeping with their old ways of working or functioning [26]. Users are seen as “technophobic, resistant and uncooperative”, therefore an obstacle to modernization [27]. The conviction that technology is going to -or should as a matter of fact- improve things is regularly confronted to what may be called “the productivity paradox”: the disjunction between the hoped for or theoretical value of the technology (e.g., organizational efficiency or improvement of practices) and the reality [28]. Such a vision has “emptied out” the technology of its “social” dimension. Its design and implementation largely have ignored the local contingencies, social interactions and clinical cultures that are specific to each context [26]. This “technology-centered” perspective typically underestimates the tensions that may exist between the instrumental value of the technology and how its recipients perceive and realize its added-value in practice [29].

In this regard, it is essential to reflect on the organizational and practice models (e.g., workflows, interprofessional collaboration, expanded teams) to be set up or adapted so that the use of technology can be at the service of better care and services for patients. The EHR may require expanding the skills of some health care providers to perform tasks that physicians, or other health care providers, usually perform (e.g., prescription renewal by nurses and pharmacists). In addition, it may also be necessary to integrate other professional profiles into the team, such as scribes or clinical assistants (e.g., documentation and note entry, messaging triage and requests, protocolized order entry), to allow health care providers to focus more on clinical work [3, 20, 22, 30, 31]. In a perspective of an active involvement and partnership, the patient’s contribution to the health care provider’s notetaking (e.g., integrated questionnaires, patient reported outcome measures) is also one of the avenues to be explored [3, 20, 32]. .That being said, these changes should not be seen as an erosion of privileges, fields of expertise (e.g., reserved activity and professional jurisdiction) or respective levels of autonomy of health care providers. Technology simply risks being rejected if it challenges the equilibriums, dynamics and “negotiated orders” between stakeholders within the organization [4, 33, 34]. In addition, the provision of training and assistance for users, particularly by experts or colleagues, in order to support a judicious and positive use (e.g. priority management, messaging, communication with the patient) of technology is an important element in promoting its adoption [30]. In this regard, technological experts and clinical leaders are pivotal to the integration of these dynamics, especially to ensure a better clinical-technological alignment; hence the relevance of developing hybrid “clinical-informatics” training/fellowship to bridge these two worlds [35, 36]. Since the health system is a “professional bureaucracy”, it is important to adopt a participatory and inclusive approach for all stakeholders to co-construct new models of care and services involving technology [37].

Third, the quadruple aim questions the dominant paradigm where technologies lack interoperability by design since a competitive market does not promote cooperation between technology providers. For financial reasons and competitive advantages, companies are generally reluctant to cooperate with their competitors. The large number of technology providers in the market further complicates the landscape [38, 39]. Yet, there is a strong agreement that the added-value of the EHR could not be achieved without true compatibility and interoperability between different systems [38, 39]. Solving this problem implies adopting results-based certification criteria and requirements. In other words, evidence on interoperability and added-value of the technology in the real-world context of use by health care providers and patients should be required (e.g., usability, conviviality, adaptability, scalability, patient-clinician or clinician-clinician communication and relationship, data access, learning curve) [30, 39]. Although such requirements should be accompanied by incentives for technology providers to develop interoperable and compatible interfaces [30, 39], the issue of interoperability should be addressed with caution. Optimal interoperability implies large flows of information and data from different sources and of various nature. This could lead to an increase in the workload of health care providers. As mentioned previously, it is essential to identify the datasets and information that are really needed to provide quality care and services for patients.

## Conclusion

Research on the unintended consequences of EHRs, but also of other IT-based tools and systems, on health care providers suggests that a critical reconsideration of the IT strategies in health organizations and systems is needed. Focusing only on administrative processes for billing and control purposes may negatively affect organizational performance and destroy local dynamics that work well in certain environments. For Zulman et al. (2016), “deimplementing” EHRs could even “actively enhance care in many clinical scenarios” [40]. In this paper, we rather argued that the design and implementation of EHRs should deliberately emphasize the improvement of patient experience, population health and professional practices, as well as the provision of high-quality, coordinated and efficient services. This is what the quadruple aim puts at the forefront.

## Data Availability

The data supporting this study will be available upon request.

## Abbreviations

EHR: Electronic Health Record
IT: Information Technology
